# Early intervention to promote oral feeding in patients with intracerebral hemorrhage: a retrospective cohort study

**DOI:** 10.1186/1471-2377-11-6

**Published:** 2011-01-19

**Authors:** Hideaki Takahata, Keisuke Tsutsumi, Hiroshi Baba, Izumi Nagata, Masahiro Yonekura

**Affiliations:** 1 Department of Neurosurgery, National Hospital Organization, Nagasaki Medical Center, Nagasaki, Japan; 2 Department of Neurosurgery, Nagasaki University Graduate School of Biomedical Sciences, Nagasaki, Japan

## Abstract

**Background:**

Stroke is a major cause of dysphagia, but little is known about when and how dysphagic patients should be fed and treated after an acute stroke. The purpose of this study is to establish the feasibility, risks and clinical outcomes of early intensive oral care and a new speech and language therapist/nurse led structured policy for oral feeding in patients with an acute intracerebral hemorrhage (ICH).

**Methods:**

A total of 219 patients with spontaneous ICH who were admitted to our institution from 2004 to 2007 were retrospectively analyzed. An early intervention program for oral feeding, which consisted of intensive oral care and early behavioral interventions, was introduced from April 2005 and fully operational by January 2006. Outcomes were compared between an early intervention group of 129 patients recruited after January 2006 and a historical control group of 90 patients recruited between January 2004 and March 2005. A logistic regression technique was used to adjust for baseline differences between the groups. To analyze time to attain oral feeding, the Kaplan-Meier method and Cox proportional hazard model were used.

**Results:**

The proportion of patients who could tolerate oral feeding was significantly higher in the early intervention group compared with the control group (112/129 (86.8%) vs. 61/90 (67.8%); odds ratio 3.13, 95% CI, 1.59-6.15; P < 0.001). After adjusting for baseline imbalances, the odds ratio was 4.42 (95% CI, 1.81-10.8; P = 0.001). The incidence of chest infection was lower in the early intervention group compared with the control group (27/129 (20.9%) vs. 32/90 (35.6%); odds ratio 0.48, 95% CI, 0.26-0.88; P = 0.016). A log-rank test found a significant difference in nutritional supplementation-free survival between the two groups (hazard ratio 1.94, 95% CI, 1.46-2.71; P < 0.001).

**Conclusions:**

Our data suggest that the techniques can be used safely and possibly with enough benefit to justify a randomized controlled trial. Further investigation is needed to solve the eating problems that are associated with patients recovering from a severe stroke.

## Background

Stroke often alters a patient's dietary intake because of both dysphagia and impaired consciousness. Dysphagia occurs in 30-50% of conscious patients shortly after the onset of stroke [1-4]. Although swallowing difficulties generally improve naturally and quickly, in approximately 10% of patients, problems may persist for six or more months following a stroke. There are no reliable studies on the ability of unconscious patients to eat and drink because ordinary clinical tests, such as the water swallowing test and videofluoroscopic swallowing evaluations, are not applicable to patients who are unable to cooperate. Consequently, patients with impaired consciousness have been excluded from many clinical studies assessing swallowing after a stroke [5]. However, it is well known that impaired consciousness is a common symptom soon after a stroke [6]. Because patients who cannot tolerate oral feeding due to diminished consciousness are a significant group, studies that are limited to patients who can perform the water swallowing test or undergo videofluoroscopic evaluations do not reflect the full extent of eating problems after stroke.

To provide adequate nutrition, the insertion of a nasogastric tube or a percutaneous endoscopic gastrostomy may be performed in patients with impaired consciousness or severe dysphagia. However, severe dysphagia is a predictor of poor outcomes, and high incidences of chest infection and death are each associated with both methods of tube-feeding [7-9].

Intracerebral hemorrhage (ICH) accounts for only 10-20% of all strokes, but it is the most disabling form of stroke. Between one-third and one-half of patients with ICH do not survive, and only 10-20% of them regain functional independence [10,11]. A large percentage of ICH patients require nutritional supplementation during the early phase of their illness. The purpose of this study is to examine the effects of early intensive oral care and a new speech and language therapist (SLT)/nurse led structured policy for oral feeding on clinical outcomes, in terms of survival, the incidence of chest infection, the length of the hospital stay and swallowing function in acute ICH patients.

## Methods

### Study design

A before-and-after retrospective cohort study from January 2004 to December 2007 included patients who were admitted to our institution, where an early intervention program for swallowing was introduced in April 2005. This study was approved by the medical ethics committee at our institution.

### Study population

The diagnosis of ICH was based on symptoms, neurological signs and the results of a brain computed tomography (CT) scan. Magnetic resonance imaging and angiography were also routinely performed. Conventional digital subtraction angiography and/or three-dimensional CT angiography were also performed to exclude organic lesions.

The early intervention program was based on a behavioral intervention coordinated by a team, and it typically required several months for all of the team members to attain sufficient experience. Therefore, patients allocated to the early intervention group were recruited from those who were admitted after January 2006. In this period, there were 153 patients with spontaneous ICH who were admitted within three days of onset, including those who were comatose or intubated upon admission. Of the 153 screened patients, 22 who succumbed to the initial brain damage or complications directly attributable to ICH (e.g., extension of the hematoma and acute hydrocephalus) were excluded. One patient with an intraventricular hemorrhage without a parenchymal hematoma and another patient with multiple hematomas were also excluded. Therefore, 129 patients were allocated to the early intervention group. Starting in January 2004, there were 106 patients with spontaneous ICH who were admitted and discharged by March 2005. Of the 106 patients screened for the control group, thirteen patients who died from the initial brain damage, two patients with intraventricular hemorrhages and one patient with multiple hematomas were excluded, which resulted in a total of 90 patients allocated to the control group.

### Intervention

The early intervention program was primarily carried out by ward nurses under the guidance of a SLT and one of the authors of this study (HT).

#### 1) Intensive oral care

Oral care, which started within 24 hours of admission in all cases (regardless of the level of consciousness, intubation or ventilation status), involved teeth brushing and rinsing with 100 ml of water while the patient was supported in a lateral semi-sitting position. Water was carefully removed by suction to prevent the patient from accidentally swallowing it. After admission, a 5-minute-long oral care session was provided at least 3 times daily [12]. Opening and closing of the mouth and stimulation of the tongue and oral cavity were also performed. After oral feeding was initiated, oral care was provided before and after meals and before bedtime.

Before the initiation of oral feeding, training for sitting, proper relaxation and the placement of the neck in the chin-tuck position were performed daily by physical therapists and ward nurses.

#### 2) Commencement of oral feeding and behavioral intervention

Patients were considered eligible to commence trials of oral feeding when they regained consciousness (spontaneous eye opening), were afebrile (temperature < 38°C), and were able to maintain a supported sitting position and follow simple commands, such as releasing and clasping the examiner's hand or opening and closing their mouth [13]. The patients were then screened for dysphagia with a food test that consisted of eating pudding or jelly [14]. A fiber-optic endoscopic swallowing evaluation and a videofluoroscopic evaluation were performed only in selected patients with a long fasting period, advanced age, a previous history of stroke or a poor food test score. Specialized training from SLTs was also provided for patients with severe dysphagia.

To initiate oral feeding, a diet of the appropriate texture (jelly or puree) was chosen, and the patient was fed following postural adjustment (usually in the semi-sitting position with the chin tucked in) while oxygen saturation levels were monitored. Trained nurses performed the intervention during feeding for a maximum of one hour (average = 30 minutes) three times per day. The patient's posture and/or the food texture were adjusted according to the patient's response.

After introducing the program, the interventions described above were performed in all of the patients who could not initially respond at the time of admission. Behavioral interventions after screening with the food test were also performed in conscious patients, except for those with minimal neurological deterioration. Nutritional intake was monitored weekly by members of a nutritional support team to ensure adequate nutrition during the patients' hospital stays.

#### 3) Procedures in the control group

Prior to the introduction of this program, oral care with brushing and rinsing was provided once per day to all cases, regardless of the level of consciousness and intubation status. The timing of commencing oral feeding was left to the attending physician's discretion. Upon commencement of oral feeding, the repetitive saliva-swallowing test (dry swallowing test) and the 3-ml water-swallowing test were used as screens only for those with a long fasting period [15]. Diet modification and posture adjustment were done in the same way as in the early intervention group.

### Data collection and outcome assessment

Data, including the laboratory test results and imaging findings, were extracted from electronic medical charts (EGMAIN-EX, Fujitsu Co. Ltd., Tokyo, Japan). The hematoma volume was estimated using the ABC/2 formula [16]. The severity at the time of admission was evaluated using the Glasgow Coma Scale (GCS). The diagnosis of chest infection was based on three or more of the following variables: fever (> 38°C), a productive cough with purulent sputum, an abnormal respiratory examination (tachypnea > 22 breaths/minute, inspiratory crackles and/or bronchial breathing), an abnormal chest radiograph, arterial hypoxemia (PO_2 _< 9.3 kPa) and the isolation of a relevant pathogen (positive Gram stain or culture) [3]. Antibacterial drug use was assessed by a vial count that the patient had used for the chest infection and its sequelae. The functional outcome at the time of discharge was assessed with the modified Rankin Scale (mRS) and the Glasgow Outcome Scale (GOS); an mRS score of 5-6 and a GOS score of 1-2 were defined to be unfavorable outcomes.

Swallowing at the time of discharge was retrospectively assessed with the Functional Oral Intake Scale (FOIS) based on the medical records on food intake and the need for nutritional supplementation (Table 1) [17]. Patients free from nutritional supplementation (FOIS score 4-7) were classified as able to eat.

**Table 1:** Functional Oral Intake Scale (FOIS) [17]

Level 1:	Nothing by mouth.
Level 2:	Tube dependent with minimal attempts of food or liquid.
Level 3:	Tube dependent with consistent oral intake of food or liquid.
Level 4:	Total oral diet of a single consistency.
Level 5:	Total oral diet with multiple consistencies, but requiring special preparation or compensations.
Level 6:	Total oral diet with multiple consistencies without special preparation, but with specific food limitations.
Level 7:	Total oral diet with no restrictions.

### Statistical analysis

Associations between the baseline characteristics and the clinical variables in the two groups were assessed with the χ^2 ^test or Fisher's exact test for categorical variables and Student's *t*-test for quantitative data. A logistic regression analysis was also applied to identify the clinical variables that were significantly associated with oral intake without nutritional supplementation (FOIS score 4-7). Kaplan-Meier curves were used to estimate the cumulative rate of nutritional supplementation-free survival (FOIS score ≥ 4) and were compared with the results of the log-rank statistics. A multivariate Cox proportional hazard model was used to estimate the adjusted hazard ratio. Statistical processing was performed using the GraphPad Prism 4 (GraphPad Software Co. Ltd, CA, USA) and HALBAU7 (CMIC Co. Ltd, Tokyo, Japan) software programs.

## Results

There were no significant differences in the demographics, GCS score upon admission, hematoma location or hematoma volume between the two groups. Patients with a previous history of stroke or chronic kidney disease that required hemodialysis were equally distributed. No patients were undergoing tube-feeding before admission (Table 2).

**Table 2:** Baseline characteristics

	Control n = 90	Early intervention n = 129	P value
Demographics			
Age, years (SD)	68.0 (12.7)	69.2 (11.7)	0.646
Female, n (%)	40 (44.4)	51 (39.5)	0.468

Severity, n (%)			
GCS 13-15	51 (56.7)	86 (66.7)	
GCS 9-12	20 (22.2)	28 (21.7)	
GCS 3-8	19 (22.2)	15 (11.6)	0.141

Hematoma location, n (%)			
Supratentorial	68 (75.6)	109 (84.5)	0.098
Side of hematoma, n (%)			
Right	30/68(44.1)	48/109 (44.0)	0.992
Hematoma volume, ml (SD)			
Supratentorial	33.0 (29.5)	26.4 (28.8)	0.147
Infratentorial	14.5 (14.0)	13.2 (10.6)	0.739

Major comorbid illness, n (%)			
History of stroke	22 (24.4)	33 (25.6)	0.849
CKD with HD	5 (5.6)	9 (7.0)	0.672
Dementia	4 (4.4)	7 (5.4)	1.000
History of chest disease	1 (1.1)	6 (4.7)	0.141
Current smoke	17 (18.9)	34 (26.4)	0.198

Surgical procedure, n (%)			
Total	41 (45.6)	33 (25.6)	0.002
Trepanation	15 (16.7)	8 (6.2)	0.013
Craniotomy	26 (28.9)	25 (19.4)	0.101

Data are given as n (%) or mean (SD). Chest disease includes pneumonia, Chronic Obstructive Pulmonary Disease, asthma and lung tumor.

Abbreviations: GCS, Glasgow Coma Scale; CKD, chronic kidney disease; HD, hemodialysis.

Univariate comparisons showed that the proportion of patients with a FOIS score of 4-7 was significantly higher in the early intervention group compared with the control group (86.8% (112/129) vs. 67.8% (61/90); odds ratio 3.13, 95% CI, 1.59-6.15; P < 0.001) (Table 3). The incidence of chest infection was lower in the early intervention group (20.9% (27/129) vs. 35.6% (32/90); odds ratio 0.48, 95% CI, 0.26-0.88; P = 0.016), and the use of antibacterial drugs decreased markedly in the early intervention group, from 16.3 to 6.1 vials per patient (P = 0.009).

**Table 3:** Univariate comparisons

	Control n = 90	Early intervention n = 129	P value	Odds ratio (95% CI)
**Primary Outcome, n (%)**				
Death	7 (7.8)	2 (1.6)	0.034	0.19 (0.04-0.92)

FOIS 1	17 (18.9)	8 (6.2)		
FOIS 2, 3	5 (5.6)	7 (5.4)		

FOIS 4-6 FOIS 7	46 (51.1) 15 (16.7)	74 (57.4) 38 (29.5)	0.001	3.13 (1.60-6.15)

**Secondary Outcome**				
Infection, n (%)				
Any infection	36 (40.0)	43 (33.3)	0.312	0.75 (0.43-1.31)
Chest infection	32 (35.6)	27 (20.9)	0.016	0.48 (0.26-0.88)

Antibacterial drugs, vial/patient (SD)	16.3 (39.6)	6.1 (15.6)	0.009	

Length of hospital stay, days (SD)	44.2 (27.2)	31.9 (19.8)	< 0.001	

Functional outcome, n (%)				
(favorable)				
mRS 0-2	17 (18.9)	31 (24.0)	0.366	1.36 (0.70-2.64)
GOS 4-5	18 (20.0)	42 (32.6)	0.040	1.93 (1.02-3.64)

(unfavorable)				
mRS 5-6	48 (53.3)	51 (39.5)	0.044	0.57 (0.33-0.99)
GOS 1-2	19 (21.1)	7 (5.4)	< 0.001	0.21 (0.09-0.54)

Data are given as n (%) or mean (SD).

Abbreviations: FOIS, Functional Oral Intake Scale; mRS, modified Rankin Scale; GOS, Glasgow Outcome Scale.

The overall mortality rate in the screened patients was 18.9% (20/106) in the control period and 15.7% (24/153) in the intervention period, which were not significantly different (odds ratio 0.80, 95% CI, 0.42-1.54; P = 0.503). There were seven (7.8%) fatal cases in the control group, as follows: two cases of pneumonia, two pulmonary embolisms, one respiratory failure other than pneumonia, one acute myocardial infarction and one bladder hemorrhage. In the intervention group, there were two (1.6%) fatal cases; one patient died of pneumonia and one patient died of an aortic aneurysm rupture. Although no difference was observed in the overall mortality rate, both the proportion of patients with an unfavorable functional outcome and the mortality rate among the ICH survivors were significantly decreased in the early intervention group.

As inferred from the logistic regression analysis, early intervention was significantly correlated with a favorable oral feeding outcome (odds ratio 4.42, 95% CI, 1.81-10.8; P = 0.001) (Table 4). Although a larger number of patients in the control group received surgical procedures, surgery was not correlated with swallowing function after an adjustment for baseline imbalances.

**Table 4:** Adjusted odds ratio: results of a logistic regression analysis

Variables	Odds ratio (95% CI)	*P *value
Age (years)	0.93 (0.89-0.97)	< 0.001
Sex (female)	1.27 (0.54-3.01)	0.581
Hematoma location (infratentorial)	0.25 (0.09-0.73)	0.011
Hematoma volume (ml)	0.97 (0.95-0.99)	0.001
Severity, GCS 15-13	1.00	0.002
GCS 12-9	0.19 (0.07-0.54)	
GCS 8-3	0.13 (0.03-0.47)	
Surgery	1.80 (0.57-5.69)	0.316
Previous history of stroke	0.75 (0.30-1.89)	0.545
Early intervention	4.42 (1.81-10.8)	0.001

Abbreviations: GCS, Glasgow Coma Scale.

**Figure 1:**
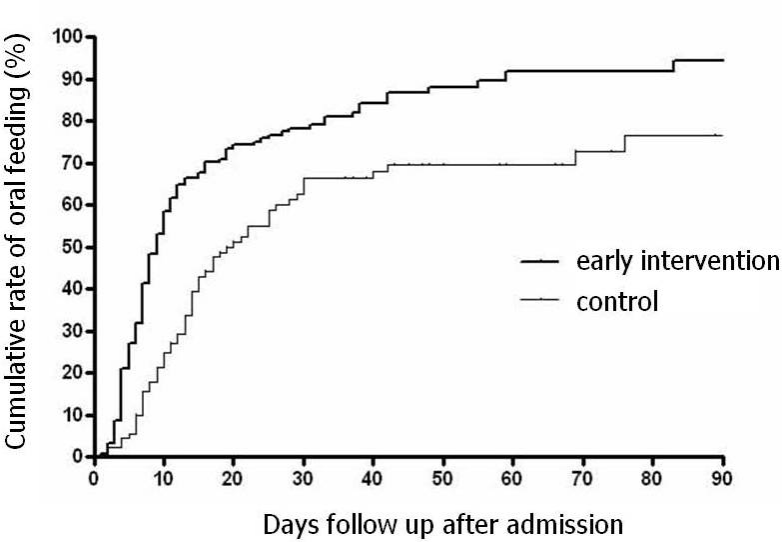
**Time to attain oral feeding**. Hazard ratio 1.94 (95% CI, 1.46-2.71; log-rank test, P < 0.001)

The Kaplan-Meier method was used to calculate the cumulative rate of nutritional supplementation-free survival, and a significant difference was observed between the two groups (hazard ratio 1.94, 95% CI, 1.46-2.71; log-rank test, P < 0.001) (Figure 1). The adjusted hazard ratio using a Cox proportional hazard model for early intervention was 2.07 (95% CI, 1.50-2.86; P < 0.001) (Table 5).

**Table 5:** Adjusted hazard ratio; results of an analysis with a Cox hazard proportional model

Variables	Hazard ratio (95% CI)	*P *value
Age (years)	0.98 (0.96-0.99)	< 0.001
Sex (female)	0.85 (0.61-1.18)	0.254
Hematoma location (infratentorial)	0.58 (0.38-0.89)	0.121
Hematoma volume (ml)	0.98 (0.97-0.99)	< 0.001
Severity, GCS 15-13	1.00	< 0.001
GCS 12-9	0.46 (0.30-0.69)	
GCS 8-3	0.28 (0.15-0.52)	
Surgery	1.10 (0.70-1.74)	0.689
Previous history of stroke	1.06 (0.74-1.52)	0.755
Early intervention	2.07 (1.50-2.86)	< 0.001

Abbreviations: GCS, Glasgow Coma Scale.

## Discussion

Our study showed that a high percentage of acute ICH patients were able to eat after intensive oral care that was followed by an early behavioral intervention, and our new policy for oral feeding did not increase the incidence of chest infection, unfavorable functional outcomes or death.

There have been no major studies on swallowing in patients with ICH because the incidence of ICH is much lower than that of cerebral infarction, and it is difficult to evaluate swallowing function in patients with severe impairments. Therefore, in previous studies on dysphagia following stroke, the majority of the subjects were conscious cerebral infarction patients who were able to cooperate with the clinical evaluation of swallowing [5,18]. The present study is one of the few surveys on dysphagia in patients with acute ICH and the first investigation to attempt to identify the optimal timing for the initiation of oral feeding after stroke.

Carnaby et al [19], first reported the effectiveness of early behavioral interventions in the recovery of swallowing in acute stroke patients with dysphagia. They studied 306 patients with clinical dysphagia and assessed survival on a normal diet at six months as the primary outcome. There was a better outcome with an early behavioral swallowing intervention, which included active therapy and dietary modifications. Despite differences in the study design, patient background and outcome measures, both the Carnaby study and the present study indicate that early behavioral interventions are effective in improving swallowing and reducing chest infections in acute stroke patients. In contrast to these two studies, which involved acute stroke patients, a study of patients undergoing rehabilitation by DePippo et al [20], did not find a positive outcome for the functional recovery of dysphagia, suggesting that intervention should be started even earlier.

The definition of dysphagia is different in each study, and, currently, there is no widely accepted outcome measure for swallowing [18]. The FOIS was developed by Crary et al [17], to evaluate swallowing based on nutritional status and diet texture. It is a simple scale with a high intra-rater agreement and sensitivity and has been validated by comparison with the Mann Assessment of Swallowing Ability and videofluoroscopic swallowing evaluations. The FOIS is applicable to patients with impaired consciousness, and, therefore, can potentially be used to objectively quantify swallowing in all stroke patients.

Our intervention protocol had two components, which included intensive oral care and a behavioral intervention. Intensive oral care was combined with mouth rehabilitation as a preparation for oral feeding. It has been reported that oral care reduces the incidence of pneumonia and elevates the serum concentration of substance P, a neurotransmitter that is involved in the cough and swallowing reflexes, in nursing home patients [12]. We suspect that oral care might also promote the recovery of swallowing ability in acute stroke patients by elevating the level of serum substance P and reducing the incidence of chest infections. However, the elucidation of the mechanisms that led to the recovery of swallowing in our patients will require further study.

The food test is the main screening tool for initiating oral feeding in our protocol. The importance of the clinical evaluation of dysphagia in reducing the incidence of chest infections is well recognized [21], but the measures that are used to avoid chest infections are different from those that are used to improve swallowing. Water is generally used for the clinical evaluation of swallowing, but it quickly passes the pharynx and easily causes choking even in patients with mild dysphagia. Therefore, a water swallowing test may be useful in detecting mild dysphagia in patients who are already eating but may not be appropriate for those with a long fasting period. Conversely, jelly and pudding are easier to swallow than water and are used in the food test. Although it may miss a diagnosis of mild dysphagia, this method is a more appropriate screening test for the initiation of oral feeding than the water swallowing test. The reduced incidence of chest infection and improved swallowing function in the present study could support the utility of the food test for safely determining the appropriate time to initiate oral feeding in patients with dysphagia after a stroke.

There was a significant decrease in the number of patients undergoing surgery in the early intervention group. An international study in 2005 indicated that there was no advantage to the early surgical evacuation of hematomas compared with medical treatment [22]. The updated American Heart Association/American Stroke Association guidelines for the management of ICH were released in 2007 and the routine evacuation of supratentorial ICH by standard craniotomy within 96 hours of ictus was not recommended [11]. Therefore, it was suggested that the reduction in surgical procedures results from evidence-based practices and is not associated with the outcome of oral feeding (Table 4).

Although this is a study on oral feeding, the diagnoses of dysphagia were not made before the initiation of treatment because the interventions (such as mouth rehabilitation, neck-relaxation and training for the sitting positions) preceded the formal diagnostic tests for dysphagia in our protocol. Instead of being diagnosed, the patients received both care and interventions soon after admission because they were at risk for complications related to dysphagia. In acute stroke patients, starting the care and interventions after a formal diagnosis is made may be too late because the usual clinical tests for dysphagia are not applicable to patients with severe impairments who are at a greater risk of dysphagia-related complications. Therefore, this study included all ICH patients and compared the overall results between the two groups.

There are several weaknesses in this study. It was a retrospective cohort study performed at a single center with unavoidable biases and limitations. A potential bias also arises from the retrospective application of outcome scores by non-blinded observers. A number of therapeutic interventions used in the early intervention group make it difficult to deduce which part of this treatment regime may carry the main responsibility for the result. However, these weaknesses may partly reflect the current problems in performing a randomized controlled trial (RCT) on dietary intake in severely affected stroke patients without a common measure for swallowing.

It is difficult to conduct a prospective intervention trial on daily care when stroke patients and their family members prefer more dedicated care. It is also difficult to carry out a double-blind study of behavioral intervention that takes weeks to accomplish because the patients who are involved in the study may eventually recognize that they were placed into separate control groups. This obstacle was mentioned by Carnaby et al [19], in their RCT, in which no significant differences were observed between the treatment and control groups using the log-rank test. The FOIS, which is applicable to unconscious patients, was only recently made available. These reasons are probably why there have not been any prospective, multicenter, double-blind trials of rehabilitation for dysphagia in the past [18,23]. Further studies should prospectively and separately evaluate the effects of intensive oral care and early intervention in severely affected acute stroke patients in the future.

In this study, the effects of early intervention were retrospectively compared with previous results. Thus, the follow-up period was short and inconsistent. However, the Kaplan-Meier curves obtained for our patients revealed a significant and rapid improvement in swallowing ability in the early intervention group, and the curves did not cross over. In addition, the results obtained by the univariate methods were consistent with those obtained by the multivariate methods in both the categorical and survival analyses. These consequences appear to support the efficacy of early intervention. Although there are no previous studies on swallowing dysfunction in severe ICH patients, our data on the functional independence and mortality obtained during the control period are comparable to the results reported in the literature [24]. Therefore, our data on swallowing function that were obtained in 2004 and 2005 appear to be accurate.

## Conclusions

The early initiation of oral feeding after sufficient preparation may safely improve the clinical outcomes of ICH patients, in terms of survival, the incidence of chest infection, the length of hospital stay and swallowing function. A common outcome measure for swallowing that can be applied to any patient may be vital in the study of eating problems and in the establishment of evidence-based practices for the treatment of dysphagia. The techniques used in this study can be used safely and possibly with enough benefit to justify a RCT. Further investigations and a multi-center RCT should be carried out in the future to improve oral feeding in stroke patients.

## Abbreviations

ICH: intracerebral hemorrhage; SLT: speech and language therapist; CT: computed tomography; GCS: Glasgow Coma Scale; mRS: modified Rankin Scale; GOS: Glasgow Outcome Scale; FOIS: functional oral intake scale; RCT: randomized controlled trial.

## Competing interests

Hideaki Takahata, M.D.: None

Keisuke Tsutsumi, M.D.: None

Hiroshi Baba, M.D.: None

Izumi Nagata, M.D.: None

Masahiro Yonekura, M.D.: None

## Authors' contributions

HT participated in the development and design of the study, conducted and supervised assessments and procedures within the study, reviewed and interpreted data, and participated in the development of the manuscript. KT and HB participated in the development and design of the study, conducted and supervised assessments within the study, and supervised and participated in the development of the draft. IN and MY supervised, provided critical review of the draft, and participated in the development of the manuscript. All authors read and approved the final manuscript.

## Pre-publication history

The pre-publication history for this paper can be accessed here:

http://www.biomedcentral.com/1471-2377/11/6/prepub
